# Food Insecurity Prevalence Among US Medical Students

**DOI:** 10.1001/jamanetworkopen.2025.29926

**Published:** 2025-08-29

**Authors:** Bassel M. Shanab, Pavan Khosla, Nour M. Hammad, Samuel J. Steuart, Morgan Brinker, George Sun, Arman T. Zadeh, David T. Gilbert, Brendan L. Rooney, Louisa Darko, Noah Engel, Yuxing Emily Ma, Kiara Smith, Marilyn Yamamoto, Stacy Goins, Edwin Owolo, Nathan Luzum, Madison Weigand, Caroline Montag, Mikhayla Armstrong, Christian Gonzalez, Nikita Desai, Cindy W. Leung, John S. Francis

**Affiliations:** 1 Yale University School of Medicine, New Haven, Connecticut; 2 Department of Nutrition, Harvard T.H. Chan School of Public Health, Boston, Massachusetts; 3 Harvard Medical School, Boston, Massachusetts; 4 Case Western Reserve University School of Medicine, Cleveland, Ohio; 5 Frank H. Netter School of Medicine, Quinnipiac University, North Haven, Connecticut; 6 Johns Hopkins University School of Medicine, Baltimore, Maryland; 7 Duke University School of Medicine, Durham, North Carolina; 8 Pritzker School of Medicine, University of Chicago, Chicago, Illinois; 9 University of Pittsburgh School of Medicine, Pittsburgh, Pennsylvania; 10 Harvard T.H. Chan School of Public Health, Boston, Massachusetts; 11 Office of Student Affairs, Yale University School of Medicine, New Haven, Connecticut

## Abstract

**Importance:**

Food insecurity affects nearly 13.5% of US households and has worsened after the COVID-19 pandemic. However, the prevalence, associated factors, and impact on medical students is unclear, hindering targeted interventions.

**Objective:**

To determine the prevalence of food insecurity among US allopathic medical students and identify associated sociodemographic factors to guide solutions.

**Design, Setting, and Participants:**

This survey study was conducted across 8 US medical schools. A cross-sectional survey was administered to medical students between March 03, 2023, and September 19, 2023. Data were analyzed from March 2024 to May 2025.

**Exposure:**

Sociodemographic characteristics of the participants: age, gender identity, race (African American; American Indian and Alaska Native or Native Hawaiian and Other Pacific Islander; Black; Central Asian; East Asian; Middle-Eastern or North African; South Asian; Southeast Asian; White, and other race), ethnicity, citizenship status, program of study, academic year, estimated total debt, sources of educational funding, undergraduate Pell grant recipient status, underrepresented in medicine (URiM) status, and basic needs insecurities.

**Main Outcomes and Measures:**

The primary outcome was the prevalence of food insecurity, using the US Department of Agriculture Adult Food Security Survey Module. Univariable and multivariable regression were used to measure associations with demographic factors.

**Results:**

A total of 1834 medical students (1073 cisgender women [58.5%]; median [IQR] age, 25 [24-27] years; 347 East Asian [18.9%]; 198 South Asian [10.8%]; and 907 White [49.5%]), food insecurity prevalence was 21.2% (389 individuals), with institutional prevalences ranging 16.0% to 31.9%. Compared with individuals identifying as White, food insecurity was more prevalent among medical students identifying as Black (odds ratio [OR], 2.91; 95% CI, 1.90-4.41), Southeast Asian (OR, 5.73; 95% CI, 2.43-13.81), Middle Eastern or North African (OR, 2.80; 95% CI, 1.47-5.17), and other race (OR, 2.65; 95% CI, 1.16-5.75), and food insecurity was higher among Hispanic or Latino individuals than non-Hispanic or Latino individuals (OR, 2.47, 95% CI, 1.75-3.45). Students of URiM backgrounds compared with non-URiM peers (OR, 2.45; 95% CI, 1.86-3.23) and Pell grant recipients compared with nonrecipients (OR, 3.00, 95% CI, 2.30-3.90) were more likely to be food insecure. Relative to students with parental financial support, those using private loans (OR, 15.43; 95% CI, 3.20-82.79), state scholarships (OR, 5.79; 95% CI, 1.16-23.66), school scholarships (OR, 4.06; 95% CI, 2.55-6.78), student contributions (OR, 3.40; 95% CI, 2.13-5.69), federal loans (OR, 3.29; 95% CI, 1.85-5.98), and other scholarships (OR, 3.25; 95% CI, 1.32-7.55) had higher odds of food insecurity as did those who had dependents (OR, 3.25; 95% CI, 2.25-4.68) or higher estimated debt (adjusted OR, 1.003; 95% CI, 1.002-1.005).

**Conclusions and Relevance:**

In this survey study of US medical students, the prevalence of food insecurity was nearly double the national household average, with significant disparities among students. These findings call for institutional and policy interventions to address medical students’ basic needs, ensuring the success of future physicians.

## Key Points

**Question:**

What is the prevalence of food insecurity among US medical students, and which students are at higher risk?

**Findings:**

In this survey study of 1834 medical students from 8 US medical schools, 21.2% were food insecure. Food insecurity was significantly higher among ethnic and racial minority students, students with dependents, and students with financial need; conversely, food insecurity was lower among those with parental tuition assistance.

**Meaning:**

In this survey study, more than 1 in 5 US medical students reported food insecurity, nearly double the level of US households, underscoring the need for targeted interventions to support medical students’ basic needs and promote academic success and well-being.

## Teaser

This survey study examines the prevalence of and sociodemographic factors associated with food insecurity among medical students in the US.

## Introduction

Approximately 13.5% of US households suffer from food insecurity,^1^ a public health crisis that has worsened in recent years with the cessation of expanded federal Supplemental Nutrition Assistance Program (SNAP) benefits under emergency COVID-19 pandemic declarations.^2^ With nutritious meals often being sacrificed in times of need due to time, financial, or accessibility constraints, food insecurity is a telling vital sign in the clinical setting. However, food insecurity among medical students has been scantly documented, with recent studies largely focusing on undergraduates.

Recent data suggest college campuses have higher rates of food insecurity than the average US household, estimated as being upward of 22%,^3^ with graduate student (ie, doctoral students and Master’s students) and postdoctoral trainee estimates of 17% and 13%, respectively.^4^ For graduate students, available literature has shown a spike at the onset of the COVID-19 pandemic, a concerning finding given that graduate students and postdoctoral trainees are often receiving living support at federally benchmarked levels.^5,6^ Moreover, food insecurity has been shown to be negatively associated with academic success and mental well-being in college students,^7,8^ yet the extent to which food insecurity affects medical students is largely unknown.^9^ In addition to rigorous coursework and clerkships, medical students are expected to engage in scholarly research, community service, and extracurricular activities, balancing their academic tasks and clinical work with personal responsibilities, such as caring for dependents and other household needs.^10^ Those with unmet basic needs may struggle to navigate their education and training. Students from underrepresented in medicine (URiM) and first-generation and low-income backgrounds are more likely to experience food insecurity, a trend seen in both public and private medical schools.^11,12,13^ Compounded with the ever-present challenges faced by URiM and first-generation and low-income student populations, such as struggles with exhaustion-related burnout^14^ and increased rates of leave of absence,^15^ food insecurity may exacerbate the overwhelming environment medical students face upon matriculation.

Despite widespread efforts to conduct clinical screening for food insecurity, there is a lack of comprehensive assessment on the prevalence of food insecurity among medical students, hindering the establishment of equitable interventions and inadvertently perpetuating hardship that is disproportionately concentrated among already vulnerable students. Thus, we conducted what is, to our knowledge, the first multi-institutional study to examine the prevalence of food insecurity and its associations with medical student attributes.

## Methods

### Study Sample and Data Collection

This survey study received exemption from the institutional review boards of Yale School of Medicine and partnering institutions requiring additional approval and followed the Strengthening the Reporting of Observational Studies in Epidemiology (STROBE) reporting guideline.^16^ Eight medical institutions participated in this cross-sectional survey: Case Western Reserve University School of Medicine, Duke University School of Medicine, Frank H. Netter MD School of Medicine, Harvard Medical School, Johns Hopkins University School of Medicine, University of Chicago Pritzker School of Medicine, University of Pittsburgh School of Medicine, and Yale School of Medicine. Qualtrics surveys were collected from medical students between March 03, 2023, and September 19, 2023, after written informed consent via nonprobability sampling. All enrolled medical students at each site were contacted to participate in the survey using schoolwide communication email listservs and via in-person class announcements.

Food insecurity over 12 months was assessed using the validated 10-item US Adult Food Security Survey Module by the US Department of Agriculture (USDA).^17^ Using USDA guidelines, a score of 3 or greater is indicative of low or very low food security (collectively termed food insecurity). Self-reported demographic data including age, gender identity, race, ethnicity, citizenship status, program of study (eg, medical doctorate and medical doctorate dual degree), academic year, estimated total debt, sources of educational funding, undergraduate Pell grant recipient status, and basic needs insecurities (eg, housing, grocery, academic resource, health, dependent, and transportation costs) were collected. Self-reported race (options included African American, American Indian and Alaska Native, Black, Central Asian, East Asian, Middle-Eastern or North African, Native Hawaiian and Other Pacific Islander, South Asian, Southeast Asian, White, and other [defined as any race not otherwise specified]) and ethnicity (options included Hispanic or Latino or not Hispanic or Latino) were queried in separate survey questions. Due to low sample size, American Indian and Alaska Native and Native Hawaiian or Other Pacific Islander were combined and analyzed as a single category. URiM included US citizens and permanent residents identifying as African American, American Indian, Alaska Native, Black, Native Hawaiian, Pacific Islander, or Hispanic or Latino per the American Association of Medical Colleges definition.^18^ Receipt of a Pell grant as an undergraduate student was utilized as a proxy for low-income status per previously published studies on medical students.^19^

### Statistical Analysis

Responses across the 8 institutions were harmonized into 1 dataset. Duplicate responses with identical identifiers from the same institution were removed, retaining only the initial response. Participants who did not complete the first 4-question section of the USDA Adult Food Security Survey Module were excluded (62 individuals).

Descriptive statistics, including frequencies, medians, and IQRs, were employed to characterize the sample. Differences in sample characteristics between food-secure and food-insecure individuals were assessed with univariable logistic regression. Multivariable logistic regression was also utilized, adjusting for actionable student financial attributes (age, year in medical school, US citizenship or permanent resident status, having dependents, medical education funding methods, history of Pell Grant receipt, and current estimated debt), and passed all assumption testing (eFigures 1-5 in Supplement 1).

Statistical analyses were performed in R version 4.1.1 (R Project for Statistical Computing). All tests were 2-sided, and *P* < .05 was considered statistically significant. *P* values were corrected (*q* values) for multiple comparisons using the Benjamini-Hochberg false discovery rate. Data analysis was performed from March 2024 to May 2025.

Demographic characteristics of the study cohort were compared with those available from the participating institutions’ overall study population to assess representativeness.^20^ To evaluate differences in proportions of covariates between the study population and overall population, we calculated standardized mean differences for each demographic group.^21^

## Results

### Characteristics of the Study Cohort

Of 5099 invited participants, a total of 1834 medical students completed the survey (overall response rate, 36.0%; institutional response rate range, 10.2% to 61.2%; mean [SD] rate, 36.9% [17.4%]), with an overall food insecurity prevalence (FIP) of 21.2% (389 participants; institutional prevalence range, 16.0% to 31.9%) (Table 1). The median (IQR) age was 25 (24-27) years. More than one-half of the study population was cisgender women (1073 participants [58.5%]). Of the study sample, 71 (3.9%) identified as African American; 23 (1.3%) as American Indian and Alaska Native or Native Hawaiian and Other Pacific Islander; 129 (7.0%) as Black; 5 (0.3%) as Central Asian; 347 (18.9%) as East Asian; 174 (9.5%) as Hispanic or Latino; 73 (4.0%) as Middle Eastern or North African; 198 (10.8%) as South Asian; 39 (2.1%) as Southeast Asian; 907 (49.5%) as White; and 36 (2.0%) as other race or ethnicity. Approximately 344 participants (18.8%) were classified as low-income. Sociodemographic and other student characteristics are presented in Table 1.

**Table 1.** Student Characteristics and Food Insecurity Prevalence

Characteristic		Participants, No.		Participants by food security status, No. (%)^a^		
				Insecure		Secure
All participants	1834		389 (21.2)		1445 (78.8)	
Gender identity						
Cisgender man	596		126 (21.1)		470 (78.9)	
Cisgender woman	1073		212 (19.8)		861 (80.2)	
Minoritized genders^b^	49		16 (32.7)		33 (67.3)	
Race						
African American	71		21 (29.6)		50 (70)	
American Indian, Alaska Native, Native Hawaiian, or Other Pacific Islander	23		8 (34.8)		15 (65.2)	
Black	129		44 (34.1)		85 (65.9)	
Central Asian	5		2 (40.0)		3 (60.0)	
East Asian	347		58 (16.7)		289 (83.3)	
Middle Eastern or North African	73		24 (32.9)		49 (67.1)	
South Asian	198		32 (16.2)		166 (83.8)	
Southeast Asian	39		14 (35.9)		25 (64.1)	
White	907		157 (17.3)		750 (82.7)	
Other^c^	36		13 (36.1)		23 (63.9)	
Ethnicity						
Hispanic or Latino	174		62 (35.6)		112 (64.4)	
Not Hispanic or Latino	1660		327 (19.7)		1333 (80.3)	
Underrepresented in medicine^d^	319		106 (33.2)		213 (66.8)	
Year in medical school						
1st Year	615		141 (22.9)		474 (77.1)	
2nd Year	406		88 (21.7)		318 (78.3)	
3rd Year	430		82 (19.1)		348 (80.9)	
Research or dual-degree year	119		25 (21.0)		94 (79.0)	
4th Year	264		53 (20.1)		211 (79.9)	
US citizen or permanent resident	1705		331 (19.4)		1314 (80.6)	
Medical education funding						
School scholarship	829		222 (26.8)		607 (73.2)	
State scholarship	41		20 (48.8)		21 (51.2)	
Other scholarship	143		30 (21.0)		113 (79.0)	
Student contribution	550		125 (22.7)		425 (77.3)	
Parental contribution	767		94 (12.3)		673 (87.7)	
Federal loans	880		223 (25.3)		657 (74.7)	
Private loans	244		76 (31.1)		168 (68.9)	
Research stipend	209		55 (21.2)		204 (78.8)	
Employment	126		37 (29.4)		89 (70.6)	
Other funding	93		9 (9.7)		84 (90.3)	
Has dependents	134		57 (42.5)		77 (57.5)	
Received Pell grant	344		127 (36.9)		217 (63.1)	

^a^ Percentages by row, indicating the number of participants that completed the question and identified with the characteristic.

^b^ Includes transgender man, transgender woman, gender nonconforming, agender, genderqueer, or gender not listed.

^c^ Includes any race not otherwise specified.

^d^ Calculated group including US citizens and permanent residents identifying as African American, American Indian, Alaska Native, Black, Native Hawaiian, Pacific Islander, or Hispanic or Latino per the American Association of Medical Colleges definition.

Relative to the overall study population (5099 individuals), the study cohort had a lower proportion of individuals identifying as other race (36 individuals [2.0%] vs 167 individuals [3.3%]), a smaller percentage of cisgender men (596 individuals [32.5%] vs 1834 individuals [44.6%]), and a slightly higher percentage of cisgender women (1073 individuals [58.5%] vs 1834 individuals [55.3%]). Standardized mean differences indicated negligible differences across all demographic groups, apart from cisgender men, for whom the calculated effect size was moderate (eTable in Supplement 1).^21^

### Food Insecurity by Attribute

Food insecurity was significantly more prevalent among racial and ethnic minority students and those with financial vulnerabilities. Compared with students identifying as White (FIP,  157 individuals [17.3%]), those identifying as Black (FIP, 44 individuals [34.1%]; odds ratio [OR], 2.91; 95% CI, 1.90-4.41; *P* < .001), Southeast Asian (FIP, 14 individuals [35.9%]; OR, 5.73; 95% CI 2.43-13.81; *P* < .001), Middle Eastern or North African (FIP, 24 individuals [32.9%]; OR, 2.80, 95% CI 1.47-5.17; *P* = .001), or other race (FIP, 13 individuals [33.2%]; OR, 2.65; 95% CI, 1.16-5.75; *P* = .02) had significantly higher odds of food insecurity (Table 1 and Table 2). Similarly, students identifying as Hispanic or Latino (FIP, 62 individuals [35.6%]; OR, 2.47; 95% CI, 1.75-3.45; *P* < .001) or determined to be URiM (FIP, 106 of 319 individuals [33.2%]; OR, 2.45; 95% CI, 1.86-3.23; *P* < .001) had more than twice the odds of food insecurity compared with their non-Hispanic or Latino or non-URiM peers, respectively.

**Table 2.** Associations of Student Characteristics With Food Insecurity

Characteristic	Participants by food insecurity status, No./total No. (%)		Univariable logistic regression			
	Insecure (n = 389)	Secure (n = 1445)	Participants, No.	Food insecurity, OR (95% CI)	*P* value	*q* value^a^
Age, median (IQR), y	25 (24-27)	25 (24-27)	1715	1.02 (0.99-1.05)	.17	.29
Gender identity						
Cisgender man	126/354 (35.7)	470/1364 (34.5)	1716	1.09 (0.85-1.39)	.50	.74
Cisgender woman	212/354 (60.1)	861/1364 (63.2)	1716	1 [Reference]	NA	NA
Minoritized genders^b^	16/354 (4.5)	33/1364 (2.4)	1716	1.89 (0.93-3.63)	.07	.12
Race						
African American	21/323 (6.5)	50/1297 (3.9)	1620	1.10 (0.25-3.47)	.88	.99
American Indian, Alaska Native, Native Hawaiian, or Other Pacific Islander	8/323 (2.5)	15/1297 (1.2)	1620	NR	NR	NR
Black	44/323 (13.6)	85/1297 (6.6)	1620	2.91 (1.90-4.41)	<.001	<.001
Central Asian	2/323 (0.6)	3/1297 (0.2)	1620	NR	NR	NR
East Asian	58/323 (18.0)	289/1297 (22.3)	1620	0.99 (0.69-1.38)	.93	.99
Middle Eastern or North African	24/323 (7.4)	49/1297 (3.8)	1620	2.80 (1.47-5.17)	.001	.003
South Asian	32/323 (9.9)	166/1297 (12.8)	1620	0.94 (0.61-1.43)	.79	.99
Southeast Asian	14/323 (4.3)	25/1297 (1.9)	1620	5.73 (2.43-13.81)	<.001	<.001
White			1620	1 [Reference]	NA	NA
Other^c^	157/323 (48.6)	750/1297 (57.8)	1620	2.65 (1.16-5.75)	.02	.04
Ethnicity						
Not Hispanic or Latino	267/329 (81.2)	1191/1303 (91.4)	1632	1 [Reference]	NA	NA
Hispanic or Latino	62/329 (18.8)	112/1303 (8.6)	1632	2.47 (1.75-3.45)	<.001	<.001
Underrepresented in medicine^d^						
No	221/327 (68.0)	1074/1287 (83.9)	1603	1 [Reference]	NA	NA
Yes	106/327 (33.0)	213/1287 (16.1)	1603	2.45 (1.86-3.23)	<.001	<.001
Year in medical school						
1st Year	141/389 (36.2)	474/1445 (32.8)	1834	1.12 (0.70-1.84)	.65	.87
2nd Year	88/389 (22.6)	318/1445 (22.0)	1834	1.04 (0.64-1.74)	.88	.99
3rd Year	82/389 (21.1)	348/1445 (24.1)	1834	0.89 (0.54-1.49)	.64	.87
Research or dual-degree year	25/389 (6.4)	94/1445 (6.5)	1834	1 [Reference]	NA	NA
4th Year	53/389 (13.6)	211/1445 (14.6)	1834	0.94 (0.56-1.63)	.83	.99
US citizen or permanent resident						
No	18/349 (5.2)	42/1356 (3.1)	1705	1 [Reference]	NA	NA
Yes	331/349 (94.8)	1314/1356 (96.9)	1705	0.59 (0.34-1.06)	.07	.12
Medical education funding						
School scholarship	222/343 (64.7)	607/1318 (46.1)	1661	4.06 (2.55-6.78)	<.001	<.001
State scholarship	20/343 (5.8)	21/1318 (1.6)	1661	5.79 (1.16-23.66)	.02	.04
Other scholarship	30/343 (8.7)	113/1318 (8.6)	1661	3.25 (1.32-7.55)	.007	.02
Student contribution	125/343 (36.4)	425/1318 (32.2)	1661	3.40 (2.13-5.69)	<.001	<.001
Parental contribution	94/343 (27.4)	673/1318 (51.1)	1661	1 [Reference]	NA	NA
Federal loans	223/343 (65.0)	657/1318 (49.8)	1661	3.29 (1.85-5.98)	<.001	<.001
Private loans	76/343 (22.2)	168/1318 (12.7)	1661	15.43 (3.20-82.79)	<.001	.002
Research stipend	55/343 (16.0)	204/1318 (15.5)	1661	1.42 (0.57-3.25)	.42	.65
Employment	37/343 (10.8)	89/1318 (6.8)	1661	NR	NR	NR
Other funding	9/343 (2.6)	84/1318 (6.4)	1661	0.40 (0.02-2.02)	.38	.62
Has dependents						
No	285/342 (83.3)	1252/1329 (94.2)	1671	1 [Reference]	NA	NA
Yes	57/342 (16.7)	77/1329 (5.8)	1671	3.25 (2.25-4.68)	<.001	<.001
Received Pell grant						
No	216/343 (63.0)	1106/1323 (83.6)	1666	1 [Reference]	NA	NA
Yes	127/343 (37.0)	217/1323 (16.4)	1666	3.00 (2.30-3.90)	<.001	<.001
Estimated debt, median (IQR), $	65 000 (20 000-150 000)	36 000 (0-100 000)	1532	1.00 (1.00-1.00)^e^	<.001	<.001

Abbreviations: OR, odds ratio; NA, not applicable; NR, not reported for unreliable estimates with small sample size; .

^a^ False discovery rate correction for multiple testing.

^b^ Includes transgender man, transgender woman, gender nonconforming, agender, genderqueer, or gender not listed.

^c^ Includes any race not otherwise specified.

^d^ Calculated variable including US citizens and permanent residents identifying as African American, American Indian, Alaska Native, Black, Native Hawaiian, Pacific Islander, or Hispanic or Latino per the American Association of Medical Colleges definition.

^e^ More specifically, OR 1.003 (95% CI, 1.002-1.004).

There were no significant associations of food insecurity with age or year in medical school. While the point estimate for US citizenship and permanent resident status was small in magnitude (FIP, 331 of 1705 individuals [19.4%]; OR, 0.59, 95% CI, 0.34-1.06; *P* = .07) and the point estimate for minoritized gender identity, compared with cisgender woman identity was large in magnitude (FIP, 16 of 49 individuals [32.7%]; OR 1.89; 95% CI, 0.93-3.63, *P* = .07), these findings did not reach statistical significance.

Financial factors demonstrated large-magnitude associations with food insecurity. Compared with students receiving parental financial support (FIP, 94 of 767 individuals [12.3%]), those who relied on private loans (FIP, 76 of 244 individuals [31.1%%]; OR, 15.43; 95% CI, 3.20-82.79; *P* < .001), state scholarships (FIP, 20 of 41 individuals [48.8%]; OR, 5.79; 95% CI, 1.16-23.66; *P* = .02), school scholarships (FIP, 222 of 829 individuals [26.8%]; OR, 4.06; 95% CI, 2.55-6.78; *P* < .001), federal loans (FIP, 223 of 880 individuals [25.3%]; OR, 3.29; 95% CI, 1.85-5.98; *P* < .001), student contributions (FIP, 125 of 550 individuals [22.7%]; OR, 3.40; 95% CI, 2.13-5.69; *P* < .001), or other scholarships (FIP, 30 of 143 individuals [21.0%]; OR, 3.25; 95% CI, 1.32-7.55; *P* = .007) were significantly more likely to experience food insecurity. Students with dependents (FIP, 57 of 134 individuals [42.5%]; OR, 3.25; 95% CI, 2.25-4.68; *P* < .001) and those who had received an undergraduate Pell grant (FIP, 127 of 344 individuals [36.9%]; OR, 3.00; 95% CI, 2.30-3.90; *P* < .001) also had elevated odds of food insecurity (Table 1 and Table 2). Estimated educational debt was significantly higher among students experiencing food insecurity (median [IQR], $65 000 [$20 000-$150 000] vs $36 000 [$0-$100 000]; *P* < .001) (Table 2).

### Multivariable Modeling of Associations With Food Insecurity

The results of the multivariable logistic regression model are shown in Table 3. Students with dependents had significantly higher odds of food insecurity compared with those without dependents (adjusted OR [aOR], 2.55; 95% CI, 1.67-3.86; *P* < .001). Similarly, students who had received a Pell grant were more likely to experience food insecurity than those who had not (aOR, 2.31; 95% CI, 1.71-3.12; *P* < .001). Compared with students whose medical education was funded in part or in full through parental contributions, students utilizing private loans (aOR, 9.00; 95% CI, 1.71-52.24; *P* = .009), school scholarships (aOR, 2.74; 95% CI, 1.60-4.96; *P* < .001), federal loans (aOR, 2.06; 95% CI, 1.07-4.08; *P* = .03), and other scholarships (aOR, 2.77, 95% CI, 1.03-7.45; *P* = .04) had significantly higher odds of food insecurity. Employment was not included in the final model due to unreliable estimates based on small sample size. Estimated debt was positively associated with food insecurity (aOR, 1.003; 95% CI, 1.002-1.005; *P* < .001). Other characteristics such as age, year in medical school, and citizenship status remained unassociated with food insecurity in the adjusted model.

**Table 3.** Multivariable Associations of Student Financial Characteristics With Food Insecurity

Characteristic	Multivariable logistic regression		
	Food insecurity, OR (95% CI)	*P* value	*q* value^a^
Age	1.00 (0.95-1.04)	.94	.98
Year in medical school			
1st Year	1.47 (0.81-2.78)	.22	.38
2nd Year	1.26 (0.69-2.40)	.47	.65
3rd Year	0.97 (0.53-1.85)	.93	.98
Research or dual-degree year	1 [Reference]	NA	NA
4th Year	0.97 (0.51-1.89)	.92	.98
US citizen or permanent resident			
No	1 [Reference]	NA	NA
Yes	0.68 (0.33-1.52)	.32	.48
Has dependents			
No	1 [Reference]	NA	NA
Yes	2.55 (1.67-3.86)	<.001	<.001
Medical education funding			
School scholarship	2.74 (1.60-4.96)	<.001	.002
State scholarship	3.74 (0.68-17.00)	.10	.20
Other scholarship	2.77 (1.03-7.45)	.04	.10
Parental contribution	1 [Reference]	NA	NA
Federal loans	2.06 (1.07-4.08)	.03	.09
Private loans	9.00 (1.71-52.24)	.009	.03
Employment	NR	NR	NR
Other funding	0.27 (0.01-1.54)	.23	.38
Received Pell grant			
No	1 [Reference]	NA	NA
Yes	2.31 (1.71-3.12)	<.001	<.001
Estimated debt	1.00 (1.00-1.00)^b^	<.001	<.001

Abbreviations: OR, odds ratio; NA, not applicable; NR, not reported for unreliable estimates with small sample size.

^a^ False discovery rate correction for multiple testing.

^b^ More specifically, OR, 1.003 (95% CI, 1.002-1.005).

## Discussion

To our knowledge, this is the first multi-institutional survey study to assess food insecurity among US medical students. Our findings show that more than 1 in 5 medical students experience food insecurity, nearly double the national household average.^1^ Students identifying as ethnic or racial minority groups or with socioeconomic burden were at higher risk of food insecurity. Compared with students whose medical education was funded in part or in full through parental contributions, those who reported financing their medical education with school scholarships, federal loans, or private loans were also more likely to be food insecure, as were students who had higher debt or had dependents. Similarly, Black, Hispanic or Latino, and URiM students, compared with White, non–Hispanic or Latino, and non-URiM peers, respectively, were more likely to experience food insecurity.

Our findings substantiate single-institution studies that have noted similar rates as well as ethnoracial and socioeconomic associations with food insecurity among medical students—both before^11,13^ and during^12^ the COVID-19 pandemic. However, given the limited food insecurity research on medical student populations, our study illuminates the pervasive nature of food insecurity irrespective of medical school campus in our current day. Quite well-documented in the undergraduate student population, food insecurity has known negative associations with student academic performance and physical and mental health,^22^ yet, in medical students, few studies have been able to examine the associations of food insecurity with well-being and academic performance.^9,11^ Further work is needed with particular focus on medical students given their higher prevalence of depression and burnout coupled with overwhelming educational debt.^23,24,25^

Lacking established outcomes on food-insecure medical students of any one background, previous literature on medical students who are from URiM or first-generation and low-income backgrounds has shown the detrimental impact of unmet basic needs on student performance and well-being.^26,27,28,29^ Not only are these historically marginalized students more likely to be food insecure, but they also face higher rates of exhaustion-related burnout,^14^ microaggressions,^30^ discrimination,^31^ leaves of absence,^32^ attrition,^33^ and nonplacement into residency programs.^34^ For illustration, qualitative studies have alluded to the dehumanizing impact such food insecurity places on medical students and trainees.^27,33^ As the next generation of clinicians, these future physicians should be supported; at the very least, their basic needs must be met.

Remarkably, institutions may be able to act in a timely manner and accordingly lessen undue hardship associated with food insecurity. Our study found that students with dependents or receiving school scholarships, federal loans, or private loans were more likely to be food insecure, potentially reflecting gaps between estimated cost of living or student rationing of loans. In particular, with the continued rise in inflation of food and shelter costs relative to lagging student financial aid packages, inflexible costs such as rent or dependent financial obligations may be prioritized over food, forcing difficult decisions in the amount and quality of food a student can access on a limited budget.^34,35^ In either case, there are opportunities to engage students via financial aid counseling (eg, money management or revision of their budget to match their true cost of living for those students with dependents). As cost of attendance is notoriously underestimated by universities, more financial support for students is warranted.^36^ In conducting a basic needs assessment of their student body, medical schools may be able to appropriately implement interventions to best support their students. Cerasani et al^37^ have created an effective curriculum for faculty and medical education administrators to identify and address unmet basic needs among medical students; it can be readily adapted into faculty development workshops and updated with local (ie, university and municipality), state, and federal resources for students in need of access.

To illustrate, 1 of the 8 medical schools in our study developed a student-led taskforce that worked together with their school’s administration to pilot various initiatives primarily revolving around food insecurity and transportation inaccessibility, including a dining points program, wherein eligible students receive renewable credit for use at the medical school cafeteria; a hospital meal card program for preclinical and clinical students in need for use at the hospital cafeteria; a transit pass program for all students to use public bus transportation; and a rideshare reimbursement program for clerkship students for travel to and from off-campus clinical sites. Tailored programs like these are not the only approach to unmet basic needs; community partnerships with farmer’s markets on campus, at some frequency, or community-supported agriculture produce boxes may address underlying nonsocioeconomic determinants of food insecurity for medical students such as time or accessibility constraints to affordable, nutritious foods. Various studies have shown the success of such initiatives in addressing malnutrition and food insecurity among patient populations; however, unless subsidized, it is not as effective among working-class adults given its prohibitive costs.^38^ More options can include campus programming such as food offerings or food pantries to increase the amount of food available on campus. Further work is warranted to evaluate the most cost-effective and optimal approaches.

Beyond medical school, administrators may increase awareness efforts or assist eligible in-need students with enrolling in SNAP where available. Given most medical students are able-bodied adults without dependents,^39^ a common criterion for SNAP benefit eligibility revolves around working a minimum number of hours (eg, part-time status) or status as an employee of a work-study program—the former being difficult to maintain for medical students relative to their time-intensive student obligations and the latter varying by medical school financial aid policy. State eligibility criteria vary but, given the vast disparity and deleterious effects of food insecurity on student well-being and academic potential, the USDA and the Department of Education have been working to increase awareness and access for eligible students.^40^ For some students, these SNAP benefits may alleviate financial burden associated with food cost, allowing prioritization of other financial obligations such as rent or affording academic resources (ie, US Medical Licensing Examination Step study materials).^27^

With adjustments of medical school curricula to include the social determinants of health^41,42^ and the concept of food as medicine,^43,44^ considerations on how best to help our students (as much as our patients) attain their basic needs is needed. Undeniably, medical education is a bureaucratic enterprise with many competing priorities, necessitating data to justify institutional action. In combination with institutional inertia, implementation of intensive data collection often results in survey fatigue as medical students face vying surveys in their inbox. Although we accomplished a response rate of nearly 40%, we timed our study with other important data collection efforts such as the Liaison Committee on Medical Education reaccreditation independent student analyses or the American Association of Medical Colleges own series of questionnaires at matriculation, second-year, and graduation.^45,46,47^ Therefore, we propose such data collection on basic needs of medical students no longer be siloed, but instead integrated into existing, routinely utilized surveys to reduce survey fatigue and support institutional action—similar to the University of California system with its own student experience survey, including questions focused on basic needs, conducted in alternating years.^48^ From there, campus-specific interventions may be considered and implemented. However, beyond concern for sustainability and efficacy of such short-term fixes,^49^ the underlying cause of such unmet basic need must be addressed—namely, the growing disparity between cost of living and the financial aid provided to medical students.

Without addressing the cost of living with increased financial aid, medical students, particularly the most vulnerable, are in the position of enduring unmet basic needs in silence. Qualitative studies have reflected the persistent resilience of students with remarkable lived experiences, or distance traveled^27,33^; however, a culture of isolationism should not be perpetuated as medical students grow their professional identity to help others in need. Given the observed association of high debt burden with medical student food insecurity, potentially reflecting rationing behavior by borrowers to minimize further debt, medical schools should consider ways to further support their students, across multiple degree programs, with greater scholarships or via food-centered solutions across the institution.

### Limitations

Our findings should be considered in the context of the following limitations. The cross-sectional design prevents causal interpretations of the factors associated with food insecurity. Additionally, our study was conducted primarily at private, allopathic academic medical institutions in the Northeast and Midwest, nearly all within the top 50 recipients of National Institutes of Health funding, which may limit generalizability to other settings such as public institutions or institutions of lower rankings that may have greater proportions of students with lower socioeconomic status.^50,51^ Social desirability bias, common in similar studies, may also contribute to underreporting of food insecurity in our study. Additionally, the response rate, comparable with other surveys of similar focus and population,^9,11,12^ may reflect potential nonresponse bias. Although calculating standardized mean differences for differences in proportion of student covariates for available demographics revealed negligible differences across all groups apart from cisgender men, for whom the calculated effect size was moderate (eTable in Supplement 1), future work may incorporate additional survey methodology to better elucidate nonresponse bias.

## Conclusions

In this survey study of US medical students, we established a multi-institutional prevalence of food insecurity at nearly double the US household average, with significant disparities among students. These findings underscore the food insecurity crisis on medical school campuses and call for sustainable interventions, institutionally and nationally, to meet the basic needs of medical students.

## Notes

Supplement 1. **eFigure 1.** Collinearity Assumption Testing of Food Insecurity Multivariable Regression—Overall Passed **eFigure 2.** Posterior Predictive Assumption Testing of Food Insecurity Multivariable Regression—Overall Passed **eFigure 3.** Binned Residual Assumption Testing of Food Insecurity Multivariable Regression—Overall Passed **eFigure 4.** Residual Uniformity Assumption Testing of Food Insecurity Multivariable Regression—Overall Passed **eFigure 5.** Outlier Assumption Testing of Food Insecurity Multivariable Regression—Overall Passed **eTable.** Study Cohort and Population Demographics Comparison

Supplement 2.
Data Sharing Statement

## References

[1] Rabbitt MP, Reed-Jones M, Hales LJ, Burke MP. Household food security in the United States in 2023. US Department of Agriculture. Published September 4, 2024. Accessed October 28, 2024. https://www.ers.usda.gov/publications/pub-details/?pubid=109895

[2] Richterman A, Roberto CA, Thirumurthy H. Associations between ending supplemental nutrition assistance program emergency allotments and food insufficiency. JAMA Health Forum. 2023;4(8):e232511. doi:10.1001/jamahealthforum.2023.2511 37566430 PMC10422192

[3] McKibben B, Wu J, Abelson S. New federal data confirm that college students face significant—and unacceptable—basic needs insecurity. The Hope Center for Student Basic Needs. Published August 23, 2023. Accessed July 29, 2025. https://hope.temple.edu/npsas

[4] Hammad NM, Leung CW. Food insecurity among graduate students and postdoctoral trainees. JAMA Netw Open. 2024;7(2):e2356894. doi:10.1001/jamanetworkopen.2023.56894 38376842 PMC10879943

[5] Martinez SM, Esaryk E, Chodur G, . COVID-19-related stressors exacerbate food insecurity and depressive symptoms among graduate students receiving campus basic needs services: cross-sectional findings from seven California public universities. Stress Health. 2024;40(3):e3345. doi:10.1002/smi.3345 38018278

[6] National Institute of Allergy and Infectious Diseases. NIH increases NRSA stipend Levels, shares perspective. Published May 15, 2024. Accessed July 29, 2025. https://www.niaid.nih.gov/grants-contracts/nih-ups-nrsa-stipend-levels-shares-perspective

[7] El Zein A, Shelnutt KP, Colby S, . Prevalence and correlates of food insecurity among U.S. college students: a multi-institutional study. BMC Public Health. 2019;19(1):660. doi:10.1186/s12889-019-6943-6 31142305 PMC6542079

[8] Oh H, Smith L, Jacob L, . Food insecurity and mental health among young adult college students in the United States. J Affect Disord. 2022;303:359-363. doi:10.1016/j.jad.2022.02.009 35157947

[9] Parent G, Weygandt J, Lin V, Wetherill MS, Bray N, Hartwell M. Food security among medical students at a Midwest University. *Oklahoma State Medical Proceedings*. Published online December 12, 2022.

[10] Association of American Medical Colleges. What to expect in medical school. Students & Residents. Accessed August 22, 2024. https://students-residents.aamc.org/choosing-medical-career/what-expect-medical-school

[11] DeMunter J, Rdesinski R, Vintro A, Carney PA. Food insecurity among students in six health professions’ training programs. J Stud Aff Res Pract. Published online September 8, 2020. doi:10.1080/19496591.2020.1796690

[12] Zhou AG, Mercier MR, Chan C, Criscione J, Angoff N, Ment LR. Food insecurity in medical students: preliminary data from Yale School of Medicine. Acad Med. 2021;96(6):774-776. doi:10.1097/ACM.0000000000004048 34031298

[13] Riddle ES, Niles MT, Nickerson A. Prevalence and factors associated with food insecurity across an entire campus population. PLoS One. 2020;15(8):e0237637. doi:10.1371/journal.pone.0237637 32866157 PMC7458338

[14] O’Marr JM, Chan SM, Crawford L, Wong AH, Samuels E, Boatright D. Perceptions on burnout and the medical school learning environment of medical students who are underrepresented in medicine. JAMA Netw Open. 2022;5(2):e220115. doi:10.1001/jamanetworkopen.2022.0115 35195698 PMC8867243

[15] Nguyen M, Song SH, Ferritto A, Ata A, Mason HRC. Demographic factors and academic outcomes associated with taking a leave of absence from medical school. JAMA Netw Open. 2021;4(1):e2033570-e2033570. doi:10.1001/jamanetworkopen.2020.33570 33481029 PMC7823220

[16] von Elm E, Altman DG, Egger M, Pocock SJ, Gøtzsche PC, Vandenbroucke JP; STROBE Initiative. The Strengthening the reporting of observational studies in epidemiology (STROBE) statement: guidelines for reporting observational studies. Lancet. 2007;370(9596):1453-1457. doi:10.1016/S0140-6736(07)61602-X 18064739

[17] US Department of Agriculture Economic Research Service. Food security in the US—survey tools. Updated January 8, 2025. Accessed July 29, 2025. https://www.ers.usda.gov/topics/food-nutrition-assistance/food-security-in-the-u-s/survey-tools/#household

[18] American Association of Medical Colleges. FACTS Glossary. Accessed August 4, 2025. https://www.aamc.org/data-reports/students-residents/data/facts-glossary

[19] Boatright D, Nguyen M, Hill K, . Development of a tool to measure student perceptions of equity and inclusion in medical schools. JAMA Netw Open. 2024;7(2):e240001. doi:10.1001/jamanetworkopen.2024.0001 38381434 PMC10882418

[20] American Association of Medical Colleges. 2024 FACTS: enrollment, graduates, and MD-PHD data. Accessed July 29, 2025. https://www.aamc.org/data-reports/students-residents/data/facts-enrollment-graduates-and-md-phd

[21] Cohen J. Statistical Power Analysis for the Behavioral Sciences. 2nd ed. Routledge; 1988, doi:10.4324/9780203771587.

[22] Leung CW, Farooqui S, Wolfson JA, Cohen AJ. Understanding the cumulative burden of basic needs insecurities: associations with health and academic achievement among college students. Am J Health Promot. 2021;35(2):275-278. doi:10.1177/0890117120946210 32783461 PMC8754159

[23] Rotenstein LS, Ramos MA, Torre M, . Prevalence of depression, depressive symptoms, and suicidal ideation among medical students: a systematic review and meta-analysis. JAMA. 2016;316(21):2214-2236. doi:10.1001/jama.2016.17324 27923088 PMC5613659

[24] Dyrbye L, Shanafelt T. A narrative review on burnout experienced by medical students and residents. Med Educ. 2016;50(1):132-149. doi:10.1111/medu.12927 26695473

[25] American Association of Medical Colleges. Medical student education: debt, costs, and loan repayment fact card for the class of 2023. University of California San Diego Medical School. https://medschool.ucsd.edu/_files/financial-aid/aamc-2023-fact-card.pdf

[26] Nguyen M, Shanab BM, Khosla P, . The intersection of disability, race, ethnicity, and financial background on food insecurity among medical students. Acad Med. 2025. doi:10.1097/ACM.0000000000006156 40550215

[27] Havemann C, Mason HRC, Russell RG, . Challenges facing first-generation college graduates in medical school: a qualitative analysis. JAMA Netw Open. 2023;6(12):e2347528. doi:10.1001/jamanetworkopen.2023.47528 38091039 PMC10719755

[28] Nguyen J. Improving Diversity and Inclusion for First-Generation College Graduates in Medicine. Temple University; 2021. Accessed July 29, 2025. https://scholarshare.temple.edu/items/d68ce200-a3d6-4d07-8024-448336df5f87

[29] McMichael B, Lee Iv A, Fallon B, Matusko N, Sandhu G. Racial and socioeconomic inequity in the financial stress of medical school. MedEdPublish. Published online June 13, 2022. doi:10.12688/mep.17544.2 PMC9370082 36168540

[30] Anderson N, Lett E, Asabor EN, . The Association of microaggressions with depressive symptoms and institutional satisfaction among a national cohort of medical students. J Gen Intern Med. 2022;37(2):298-307. doi:10.1007/s11606-021-06786-6 33939079 PMC8811096

[31] Teshome BG, Desai MM, Gross CP, . Marginalized identities, mistreatment, discrimination, and burnout among US medical students: cross sectional survey and retrospective cohort study. BMJ. 2022;376:e065984. doi:10.1136/bmj-2021-065984 35318190 PMC8938931

[32] Nguyen M, Chaudhry SI, Desai MM, . Rates of medical student placement into graduate medical education by sex, race and ethnicity, and socioeconomic status, 2018-2021. JAMA Netw Open. 2022;5(8):e2229243. doi:10.1001/jamanetworkopen.2022.29243 36018592 PMC9419015

[33] Ellsworth BL, Solano QP, Evans J, Bidwell SS, Byrnes M, Sandhu G. Medical students’ perception of their ‘distance travelled’ in medical school applications. Med Educ. 2024;58(2):204-215. doi:10.1111/medu.15167 37485787

[34] U.S. Bureau of Labor Statistics. 12-month percentage change, Consumer Price Index, selected categories. Accessed July 29, 2025. https://www.bls.gov/charts/consumer-price-index/consumer-price-index-by-category-line-chart.htm

[35] Association of American Medical Colleges. How grad plus loans support the physician workforce patients rely on. Published 2025. Accessed July 29, 2025. https://www.aamc.org/media/82881/download?attachment=

[36] Emrey-Arras M. Financial aid offers: action needed to improve information on college costs and student aid. US Government Accountability Office. Published December 5, 2022. Accessed July 29, 2025. https://www.gao.gov/products/gao-23-104708

[37] Cerasani M, Rrapi E, Sharma T, . Identifying and addressing basic needs insecurity among medical students: a curriculum for trainees, administrators, and faculty. MedEdPORTAL. 2022;18:11195. doi:10.15766/mep_2374-8265.11195 35071750 PMC8743318

[38] Garrity K, Krzyzanowski Guerra K, Hart H, . Local food system approaches to address food and nutrition security among low-income populations: a systematic review. Adv Nutr. 2024;15(4):100156. doi:10.1016/j.advnut.2023.100156 38616069 PMC11031423

[39] Pereira-Lima K, Plegue MA, Case B, . Prevalence of disability and use of accommodation among us allopathic medical school students before and during the COVID-19 pandemic. JAMA Netw Open. 2023;6(6):e2318310. doi:10.1001/jamanetworkopen.2023.18310 37314809 PMC10267761

[40] U.S. Department of Education. (2024, November 7). New partnership between U.S. Departments of Agriculture and Education to expand SNAP awareness and access for eligible college students. EdPrepMatters. Published November 15, 2024. Accessed July 29, 2025. https://edprepmatters.aacte.org/new-partnership-between-departments-of-agriculture-and-education-to-expand-snap-awareness-and-access/

[41] Sandhu S, Solomon L, Gottlieb L. Awareness, adjustment, assistance, alignment, and advocacy: operationalizing social determinants of health topics in undergraduate medical education curricula. Acad Med. 2023;98(8):876-881. doi:10.1097/ACM.0000000000005223 37000825

[42] Weiner S. Medical schools overhaul curricula to fight inequities. Association of American Medical Colleges. Published May 25, 2021. Accessed July 29, 2025. https://www.aamc.org/news/medical-schools-overhaul-curricula-fight-inequities

[43] Rea S, Jarodiya J, Berschback M, Levine D. Improving food insecurity education in medical school through integrative service learning. J Med Educ Curric Dev. 2022;9:23821205221096286. doi:10.1177/23821205221096286 35529176 PMC9073118

[44] Thircuir S, Chen NN, Madsen KA. Addressing the gap of nutrition in medical education: experiences and expectations of medical students and residents in France and the United States. Nutrients. 2023;15(24):5054. doi:10.3390/nu15245054 38140313 PMC10745340

[45] Association of American Medical Colleges. Matriculating student questionnaire (MSQ) FAQ. Accessed July 29, 2025. https://www.aamc.org/data-reports/students-residents/matriculating-student-questionnaire-msq-faq

[46] Association of American Medical Colleges. Year two questionnaire (Y2Q) FAQ. Accessed July 29, 2025. https://aamc.org/data-reports/students-residents/year-two-questionnaire-y2q-faq

[47] Association of American Medical Colleges. Medical school graduation questionnaire (GQ) FAQ. Accessed July 29, 2025. https://www.aamc.org/data-reports/students-residents/medical-school-graduation-questionnaire-gq-faq

[48] University of California Office of the President. Basic needs annual report. University of California. Published January 2024. Accessed July 29, 2025. https://regents.universityofcalifornia.edu/regmeet/mar24/b3attach1.pdf

[49] An R, Wang J, Liu J, Shen J, Loehmer E, McCaffrey J. A systematic review of food pantry-based interventions in the USA. Public Health Nutr. 2019;22(9):1704-1716. doi:10.1017/S1368980019000144 30834852 PMC10260889

[50] Association of American Medical Colleges. Organizational characteristics database (OCD). Accessed July 29, 2025. https://www.aamc.org/data-reports/faculty-institutions/report/organizational-characteristics-database-ocd

[51] Blue Ridge Institute for Medical Research. BRIMR Rankings of NIH Funding in 2023. Published February 6, 2024. Accessed August 28, 2024. https://brimr.org/brimr-rankings-of-nih-funding-in-2023/

