# Supplementary material for: Food Insecurity Prevalence Among US Medical Students
Source: JAMA Netw Open. 2025 Aug 29;8(8):e2529926. doi: 10.1001/jamanetworkopen.2025.29926 (PMC12397891; doi:10.1001/jamanetworkopen.2025.29926)
Supplement: Supplement 1. — eFigure 1. Collinearity Assumption Testing of Food Insecurity Multivariable Regression—Overall Passed eFigure 2. Posterior Predictive Assumption Testing of Food Insecurity Multivariable Regression—Overall Passed eFigure 3. Binned Residual Assumption Testing of Food Insecurity Multivariable Regression—Overall Passed eFigure 4. Residual Uniformity Assumption Testing of Food Insecurity Multivariable Regression—Overall Passed eFigure 5. Outlier Assumption Testing of Food Insecurity Multivariable Regression—Overall Passed eTable. Study Cohort and Population Demographics Comparison [file jamanetwopen-e2529926-s001.pdf]

Supplemental Online Content

Shanab BM, Khosla P, Hammad NM, et al. Food insecurity prevalence among US medical students. *JAMA Network Open*. 2025;8(8):e2529926.
doi:10.1001/jamanetworkopen.2025.29926

eFigure 1. Collinearity Assumption Testing of Food Insecurity Multivariable Regression—Overall Passed

eFigure 2. Posterior Predictive Assumption Testing of Food Insecurity Multivariable Regression—Overall Passed

eFigure 3. Binned Residual Assumption Testing of Food Insecurity Multivariable Regression—Overall Passed

eFigure 4. Residual Uniformity Assumption Testing of Food Insecurity Multivariable Regression—Overall Passed

eFigure 5. Outlier Assumption Testing of Food Insecurity Multivariable Regression—Overall Passed

eTable. Study Cohort and Population Demographics Comparison

This supplemental material has been provided by the authors to give readers additional information about their work.

© 2025 Shanab BM et al. *JAMA Network Open*.

eFigure 1: Collinearity Assumption Testing of Food Insecurity Multivariable Regression—Overall Passed

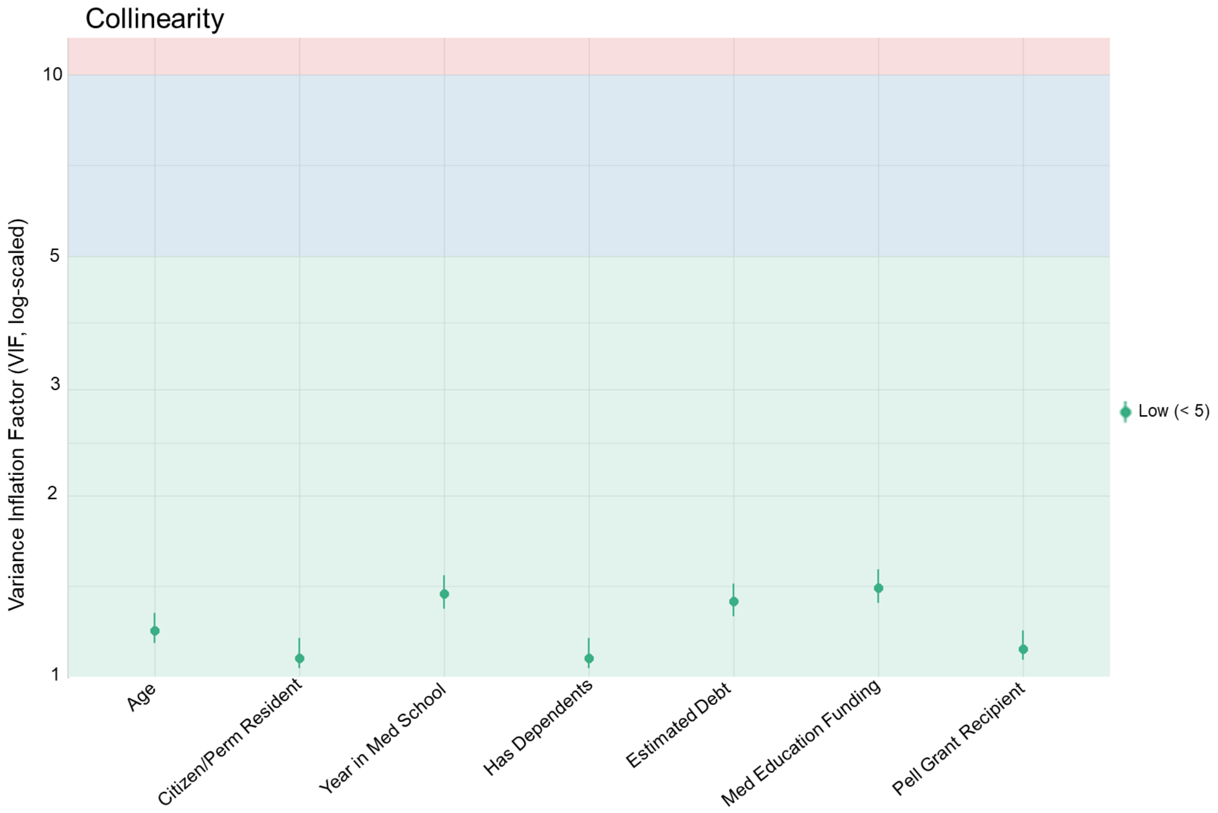

© 2025 Shanab BM et al. *JAMA Network Open*.

eFigure 2: Posterior Predictive Assumption Testing of Food Insecurity Multivariable Regression—Overall Passed

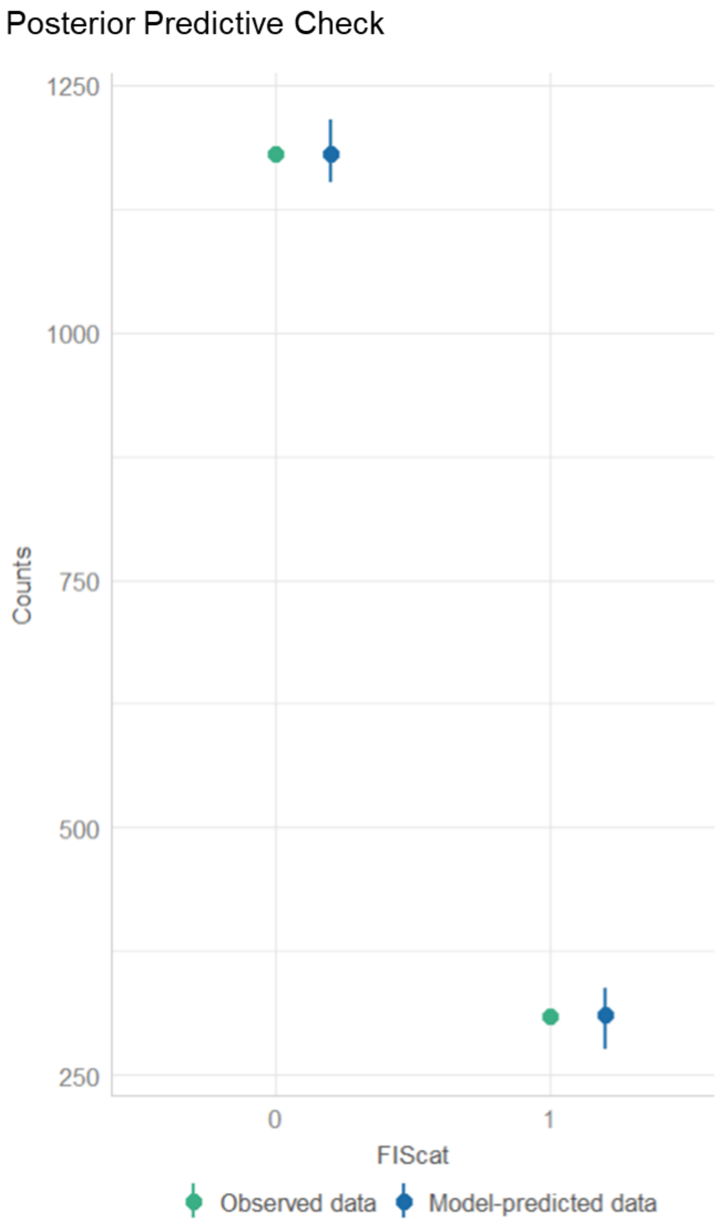

© 2025 Shanab BM et al. *JAMA Network Open*.

eFigure 3: Binned Residual Assumption Testing of Food Insecurity Multivariable Regression—Overall Passed

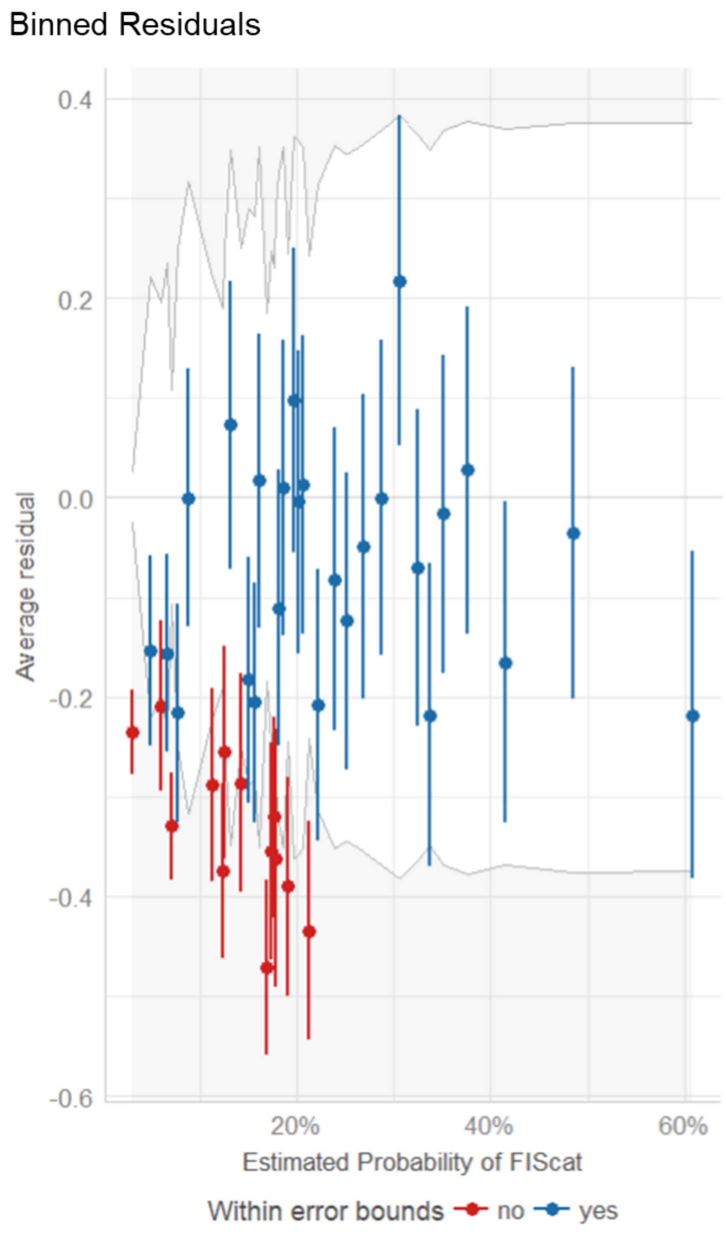

© 2025 Shanab BM et al. *JAMA Network Open*.

eFigure 4: Residual Uniformity Assumption Testing of Food Insecurity Multivariable Regression—Overall Passed

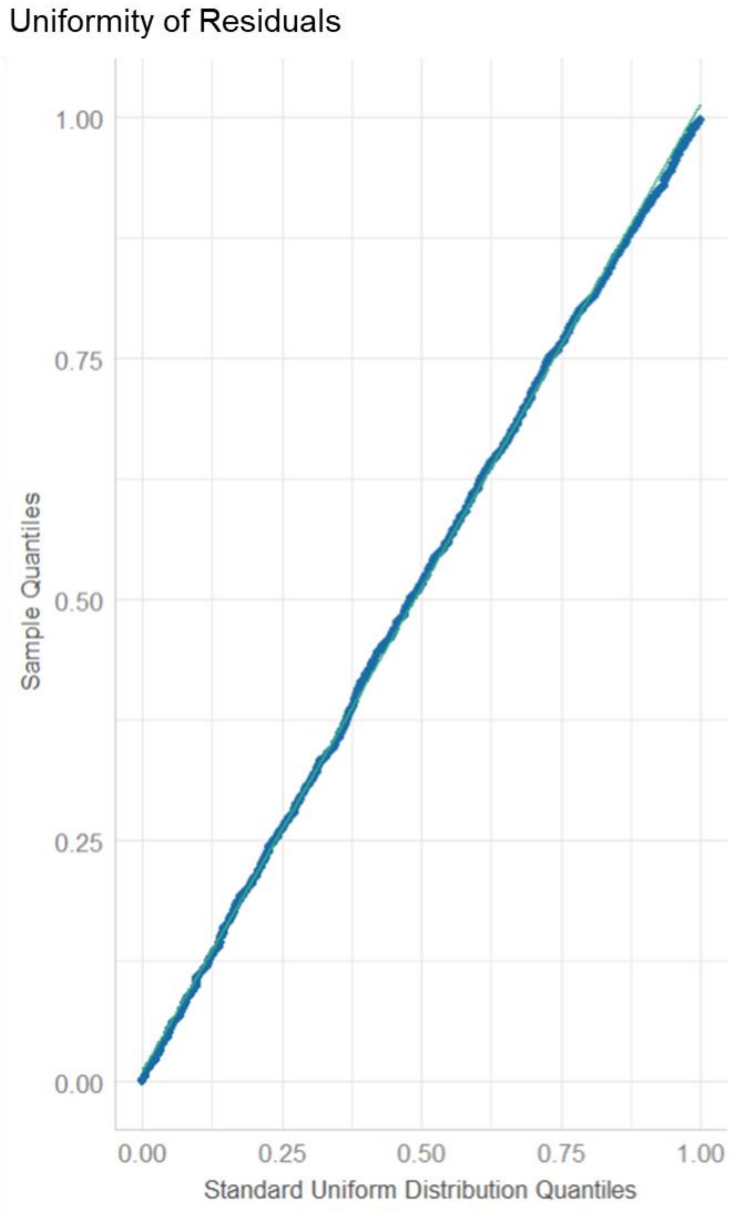

© 2025 Shanab BM et al. *JAMA Network Open*.

eFigure 5: Outlier Assumption Testing of Food Insecurity Multivariable Regression—Overall Passed

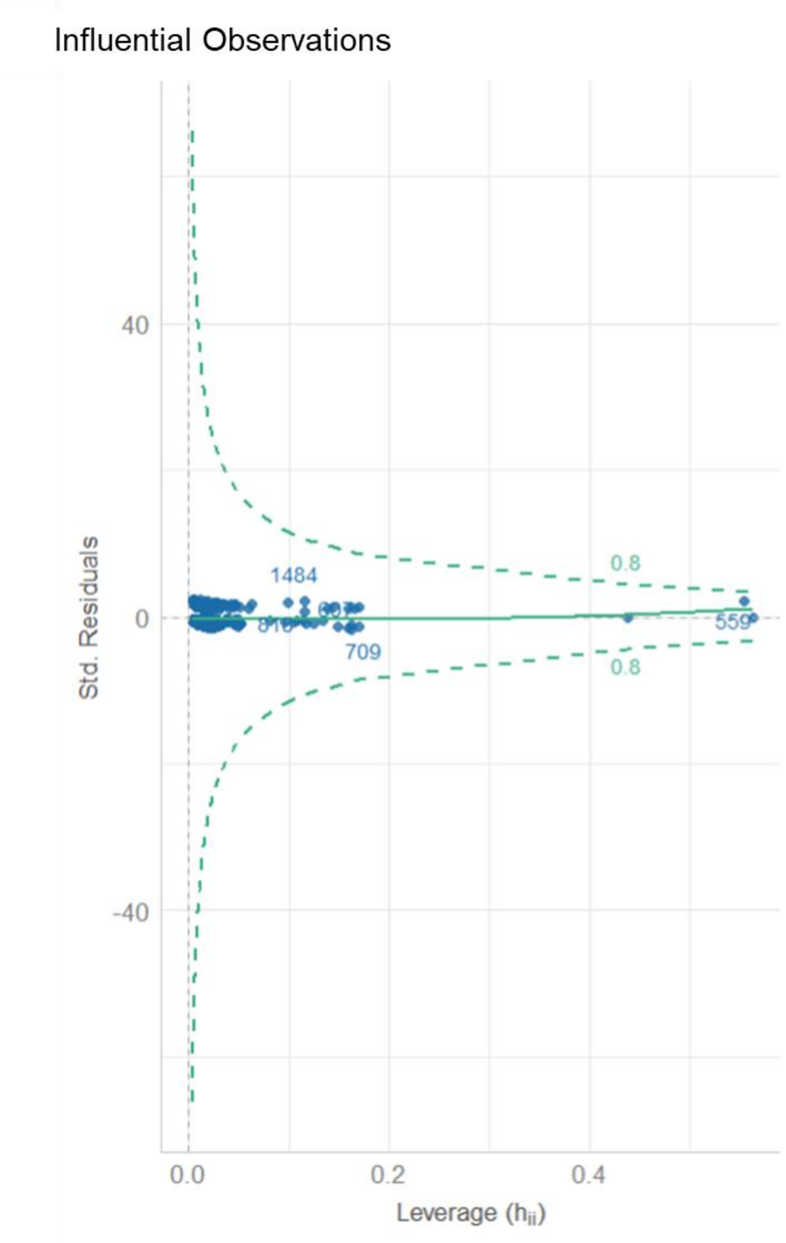

© 2025 Shanab BM et al. *JAMA Network Open*.

eTable 1: Study Cohort and Population Demographics Comparison

Category	Study N	Population N[1]	Study Total	Population Total[1]	Study Percent	Population Percent[1]	*P* value[2]	SMD[3]
**Race/ Ethnicity**							.06	
American Indian/ Alaska Native	23	72	1,834	5,102	1.3	1.4		-0.01
Asian	589	1,747	1,834	5,102	32.1	34.2		-0.04
Black/ African American	200	552	1,834	5,102	10.9	10.8		0.00
Hispanic or Latino	174	497	1,834	5,102	9.5	9.7		-0.01
Middle Eastern/ North African	73	NA[4]	1,834	NA[4]	4.0	NA[4]	NA[4]	NA[4]
White	907	2,423	1,834	5,102	49.5	47.5		0.04
Unknown	NA[4]	580	NA[4]	5,102	NA[4]	0.6	NA[4]	NA[4]
Other Race	36	167	1,834	5,102	2.0	3.3		-0.07
**Gender**							<.001	
Man	596	2,278	1,834	5,102	32.5	44.6		-0.24
Woman	1,073	2,821	1,834	5,102	58.5	55.3		0.06
Minoritized Gender	49	NA[4]	1,834	NA[4]	2.7	NA[4]	NA[4]	NA[4]

[1]Surveyed population.
[2]Chi-squared *P* value.
[3]SMD = standardized mean difference for effect size, interpreted as absolute value: <0.1 = negligible, 0.1-0.2 = small, 0.2-0.5 = moderate, >0.5 = high effect size.
[4]NA = not available for one group for comparison; not included in differences in proportion analysis.

© 2025 Shanab BM et al. *JAMA Network Open*.
