# Supplementary material for: Food Insecurity Prevalence Among US Medical Students
Source: JAMA Netw Open. 2025 Aug 29;8(8):e2529926. doi: 10.1001/jamanetworkopen.2025.29926 (PMC12397891; doi:10.1001/jamanetworkopen.2025.29926)
Supplement: Supplement 2. — Data Sharing Statement [file jamanetwopen-e2529926-s002.pdf]

Data Sharing Statement

Shanab. Food Insecurity Prevalence Among US Medical Students. *JAMA Netw Open*. Published August 29, 2025. doi:10.1001/jamanetworkopen.2025.29926

Data
Data available: No

Additional Information
Explanation for why data not available: The data collected is confidential and under various agreements between the listed study authors, the study investigators, and the participating institutions. Therefore, the data will not be made available.
